# Changing Knowledge and Attitudes Towards HIV Treatment-as-Prevention and “Undetectable = Untransmittable”: A Systematic Review

**DOI:** 10.1007/s10461-021-03296-8

**Published:** 2021-05-25

**Authors:** Jacob Bor, Charlie Fischer, Mirva Modi, Bruce Richman, Cameron Kinker, Rachel King, Sarah K. Calabrese, Idah Mokhele, Tembeka Sineke, Thembelihle Zuma, Sydney Rosen, Till Bärnighausen, Kenneth H. Mayer, Dorina Onoya

**Affiliations:** 1 Department of Global Health, Boston University School of Public Health, 801 Massachusetts Avenue, Boston, MA 02119 USA; 2 Health Economics and Epidemiology Research Office, Wits Health Consortium, Department of Internal Medicine, School of Clinical Medicine, Faculty of Health Sciences, University of Witwatersrand, Johannesburg, GP South Africa; 3 Prevention Access Campaign, New York, NY USA; 4 UCSF Institute for Global Health Sciences, University of California, San Francisco, 550 16th Street, San Francisco, CA 94158 USA; 5 George Washington University, Washington, DC USA; 6 Africa Health Research Institute, KwaZulu-Natal, South Africa; 7 University of KwaZulu-Natal, Durban, South Africa; 8 Division of Infection and Immunity, University College London, London, UK; 9 Heidelberg Institute of Global Health, Heidelberg University, Heidelberg, Germany; 10 Fenway Health Institute, Boston, MA USA; 11 Harvard Medical School, Boston, MA USA

**Keywords:** Systematic review, Treatment-as-Prevention, Undetectable = Untransmittable

## Abstract

People on HIV treatment with undetectable virus cannot transmit HIV sexually (Undetectable = Untransmittable, U = U). However, the science of treatment-as-prevention (TasP) may not be widely understood by people with and without HIV who could benefit from this information. We systematically reviewed the global literature on knowledge and attitudes related to TasP and interventions providing TasP or U = U information. We included studies of providers, patients, and communities from all regions of the world, published 2008–2020. We screened 885 papers and abstracts and identified 72 for inclusion. Studies in high-income settings reported high awareness of TasP but gaps in knowledge about the likelihood of transmission with undetectable HIV. Greater knowledge was associated with more positive attitudes towards TasP. Extant literature shows low awareness of TasP in Africa where 2 in 3 people with HIV live. The emerging evidence on interventions delivering information on TasP suggests beneficial impacts on knowledge, stigma, HIV testing, and viral suppression.

Review was pre-registered at PROSPERO: CRD42020153725

**Supplementary Information:**

The online version contains supplementary material available at 10.1007/s10461-021-03296-8.

## Resumen

Las personas en tratamiento contra el VIH con virus indetectable no pueden transmitir el VIH sexualmente (indetectable = intransmisible, U = U por sus siglas en inglés). Pero, la ciencia del tratamiento como prevención (TasP, por sus siglas en inglés) puede que no sea ampliamente comprendida por personas con y sin VIH que podrían beneficiarse. Revisamos sistemáticamente la literatura mundial sobre conocimientos y actitudes relacionados con TasP e intervenciones que proporcionan información TasP o U = U, 2008–2020. Incluimos estudios de proveedores, pacientes y comunidades de todas las regiones del mundo. Se examinaron 885 artículos y resúmenes y se identificaron 72 para su inclusión. Los estudios en entornos de ingresos altos informaron un alto conocimiento de TasP pero existen lagunas en el conocimiento sobre la probabilidad de transmisión del VIH indetectable. Un mayor conocimiento se asoció con actitudes más positivas hacia TasP. La literatura existente muestra un escaso conocimiento de TasP en África, donde viven 2 de cada 3 personas con VIH. La evidencia emergente sobre intervenciones que brindan información sobre TasP sugiere impactos positivos en el conocimiento, el estigma, las pruebas del VIH y la supresión viral.

## Introduction

Two decades ago, there was increasing recognition that plasma HIV viremia was highly associated with the risk of HIV transmission [1], and many scientists wondered if virological suppression with antiretroviral therapy (ART) could prevent transmission altogether [2]. In 2011, the HPTN-052 trial showed that ART prevented sexual HIV transmission when the person living with HIV (PLHIV) was stably virologically suppressed [3]. Since then, large cohort studies—PARTNER I (2016) and II (2019) [4, 5] and Opposites Attract (2018) [6]—have shown that the risk of sexual transmission is zero when viral load is undetectable, finding no linked transmissions in mixed-status couples across 126,000 condomless sex acts. Treatment-as-prevention (TasP) was one of the primary rationales for “Test-and-Treat”, the now widespread policy of starting ART at diagnosis regardless of CD4 count or disease stage [7], in addition to its clinical benefits [8, 9]. TasP is one of the most effective strategies to prevent HIV transmission [10], and high uptake of ART may be an effective approach to reduce HIV incidence at the population level [11].

Despite the impact of TasP on global HIV policy, information on TasP has been slower to disseminate to people with HIV and people at risk for HIV who might benefit from understanding the prevention benefits of ART [8]. In 2016, the Prevention Access Campaign launched the “Undetectable = Untransmittable” or “U = U” Campaign to spread awareness that HIV positive individuals who are virally suppressed on ART cannot sexually transmit HIV. World Health Organization, U.S. Centers for Disease Control, and National Institutes of Health leadership have publicly endorsed U = U [12] and some clinicians have called for U = U to be integrated into HIV counselling [13]. As of this writing, over 1000 organizations in over 100 countries have endorsed U = U [14]. Advocates suggest that information on TasP/U = U could reduce HIV stigma and improve uptake of HIV testing and treatment [13], which remain sub-optimal in most of the world. Despite this, national HIV programs have been slow to incorporate the concept of U = U or the benefits of TasP into HIV education initiatives [15]. Some health professionals, moreover, have been reluctant to share information on TasP/U = U, fearing unintended consequences such as unwanted pregnancy, sexually transmitted infections (STIs) [16], and HIV-acquisition if people have condomless sex without viral suppression [17]. Stigma around sexual behavior (especially same-sex sexual behavior) has also played a role in the slow dissemination of information on TasP/U = U [18].

To inform policy on TasP/U = U information dissemination, we undertook a systematic review of the global literature on beliefs about TasP/U = U. We structured our review around three questions. (1) What are current levels of awareness and knowledge of TasP/U = U among clinical providers, people with HIV in clinical care, and lay community members including people with and without HIV? (2) What are current attitudes regarding TasP/U = U and acceptability of TasP as a prevention strategy in these different populations? (3) What is the impact of interventions disseminating information on TasP/U = U on knowledge, attitudes, behaviors, and health outcomes?

## Methods

### Protocol

We followed the Preferred Reporting Items for Systematic Reviews and Meta-Analyses Protocols (PRISMA) guidelines in preparing this review [19]. We developed a review protocol, which was registered at PROSPERO: International prospective register of systematic reviews on April 4th, 2020. (https://www.crd.york.ac.uk/prospero/display_record.php?ID=CRD42020153725).

### Inclusion and Exclusion Criteria

Studies were eligible for inclusion if they were empirical studies presenting analyses of data in any of five domains pertaining to TasP/U = U: (1) awareness, (2) knowledge, (3) attitudes, (4) acceptability, and (5) impact. The impact domain included studies that presented data evaluating the effects of interventions providing accurate information regarding TasP or U = U. We included both quantitative and qualitative studies.

Our domains focused on theoretical steps leading from new information to behavioral responses, as described in the marketing [20, 21], health communications [22, 23], and technology adoption literature [24]. We defined “awareness” as having heard of TasP or U = U or having heard that HIV treatment reduces transmission risk regardless of familiarity with these specific terms. Awareness implies exposure to information about TasP or U = U, whether or not a person believes that information, and maps onto the pre-contemplation stage of the Transtheoretical model (TTM) [22]. We defined “knowledge” as being aware of TasP and holding beliefs about TasP that were consistent with the scientific literature at the time of the study. Beliefs about transmission risks with and without HIV treatment, perceived efficacy of TasP, and level of agreement with scientifically accurate statements related to TasP (including U = U) were all classified as measures of knowledge. We defined “attitudes” as encompassing emotional responses to the concepts of TasP or U = U as well as interactions of these concepts with prevailing attitudes, including HIV stigma, that may shape uptake of TasP. Following the TTM, we considered knowledge and attitudes to be formed simultaneously, not sequentially [22]. Finally, we defined “acceptability” as openness to using TasP (or recommending TasP, in the case of providers) as a strategy to prevent transmission of HIV, as well as perceived usefulness and ease of use of TasP [24]. Actual use of TasP was also coded as evidence of acceptability for the purposes of the review. These domains map onto a behavioral model in which a person becomes aware of TasP and then processes information on TasP both cognitively (knowledge) and affectively (attitudes) and evaluates whether to implement TasP in their life (acceptability).

We excluded studies that did not analyze and present data, such as opinion pieces, position statements, reviews, guidelines or recommendations, and fact sheets. We excluded studies in languages other than English. We also limited our focus to studies on the prevention of sexual transmission given current gaps in the science on TasP for injecting drug use. For studies of “impact”, we excluded studies where sharing information on TasP/U = U was not a central component of the intervention as well as studies that did not have a control group enabling inferences on causal impact.

### Information Sources

We included studies published in English between January 1st, 2008 and October 18th, 2020. Studies were included if they were published in a peer-reviewed journal indexed on PubMed, if they were presented at one of three major HIV conferences (Conference on Retroviruses and Opportunistic Infections, CROI; International AIDS Society Conference on HIV Science, IAS; International AIDS Conference, AIDS), or if they were published as a working paper or report available on Google Scholar.

### Search

We searched PubMed for articles using the following terms in the title or abstract: ("U=U" OR "U = U" OR "U Equals U" OR "Undetectable = Untransmittable" OR "Undetectable = Untransmittable" OR "Undetectable Equals Untransmittable" OR "Undetectable = Uninfectious" OR "Undetectable = Uninfectious" OR "Undetectable Equals Uninfectious" OR "TasP" OR "TASP" OR "Treatment-as-Prevention" OR "Treatment as Prevention" OR "T-as-P") AND ("HIV" OR "HIV/AIDS" OR "Human Immunodeficiency Virus"). We then searched conference abstract books for CROI (2014–2020), IAS (2010–2020), and AIDS (2018–2020) using text search functions with the same keywords. We additionally included studies referred to us through personal communication, which were identified on Google Scholar but may not have been indexed in PubMed. After our initial search returned few studies evaluating the impact of TasP/U = U interventions, we reviewed the bibliographies of those studies in the impact domain to identify others we may have missed. We also used Google Scholar’s “cited by” function to identify other, more recent studies that cited these impact studies.

### Study Selection

In a first screening step, we reviewed titles and abstracts and flagged studies for further review. In a second screening step, we assessed full text manuscripts with respect to inclusion and exclusion criteria and identified the studies to be included in the review. At each step, two authors (CF, MM) conducted the initial screening independently and a third author (JB) reviewed final selections and resolved disagreements. Duplicate records were eliminated. Where a study was presented at a conference and later published, we included just the published paper unless the data presented differed substantially. The study selection process was documented in accordance with the PRISMA-P reporting checklist.

### Data Collection and Coding

For included studies, we extracted the following information into a Google sheet database: year(s) when data were collected, location of study, study population, sampling strategy, sample size, sample characteristics, study methods, and key results. To enable standardized reporting, studies were coded according to domain (awareness, knowledge, attitudes, acceptability, or impact); study population (providers, patients, or community); and region, i.e. North America/Europe, Africa, or other (Asia, Oceania, and Central/South America). Region groupings were decided a priori based on income levels and HIV epidemiology; specific countries were also recorded for all studies. In coding study population groups, we defined providers as anyone involved professionally in clinical care for people with HIV or in HIV-related policy; we defined patients as people living with HIV who were identified through involvement in HIV care; and we defined community as people with or without HIV identified in non-clinical settings. Information on specific key populations (e.g. men who have sex with men, MSM) was extracted based on descriptors used by study authors and was not coded according to pre-assigned categories. Some studies encompassed more than one domain, population, or region. For studies on impact, we additionally extracted data on the intervention and its comparators and outcomes, following the Population-Intervention-Comparison-Outcome (PICO) framework [19]. Risk of bias was assessed for all studies by evaluating whether the sampling strategy enabled unbiased inferences about an underlying study population, and—for impact studies—whether the design enabled unbiased inferences about cause and effect.

### Data Synthesis

Our analysis proceeded in three stages. First, we assessed the coverage of the extant literature across domains, population groups, and regions in order to identify gaps. Second, we summarized quantitative measures within each of the key domains, to the extent that comparisons could be made across studies. Third, we assessed for key themes within each domain, drawing on both quantitative and qualitative studies. We did not attempt quantitative synthesis of the data into single metrics due to the diversity of study methods and lack of direct comparability of measures.

### Ethics

The study involved no human subjects, and ethics review was not required.

## Results

**Fig. 1:**
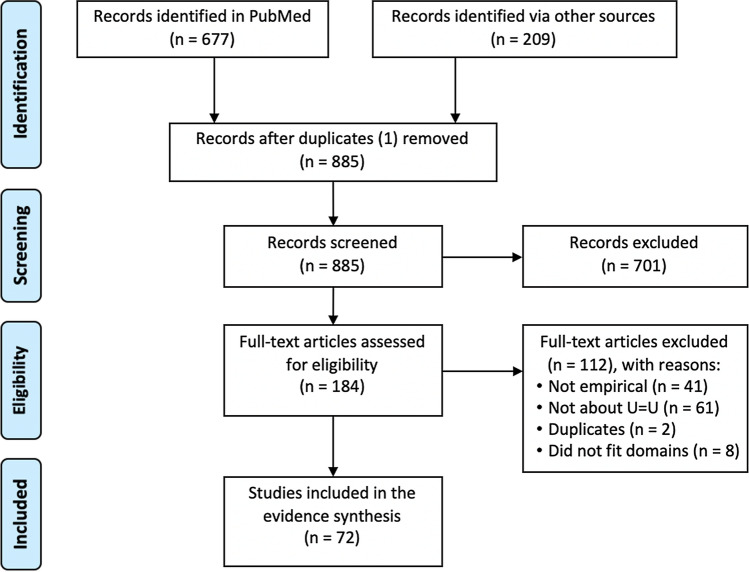
PRISMA flow diagram

Our search strategy identified 886 studies, of which 677 were from PubMed and 209 were from other sources including conference abstract books, reference searches, and personal communication (Fig. 1). After removing 1 duplicate, we had 885 studies for consideration. 701 records were excluded based on a primary screen of titles and abstracts. After screening the remaining full texts and conference abstracts, 41 were excluded because they were not empirical studies, 69 were excluded because they were not about TasP/U = U or did not address the domains of awareness, knowledge, attitudes, acceptability, or impact of TasP/U = U information; 2 were excluded because they were duplicates. Our final review included 72 studies.

### Description of Included Studies

**Fig. 2:**
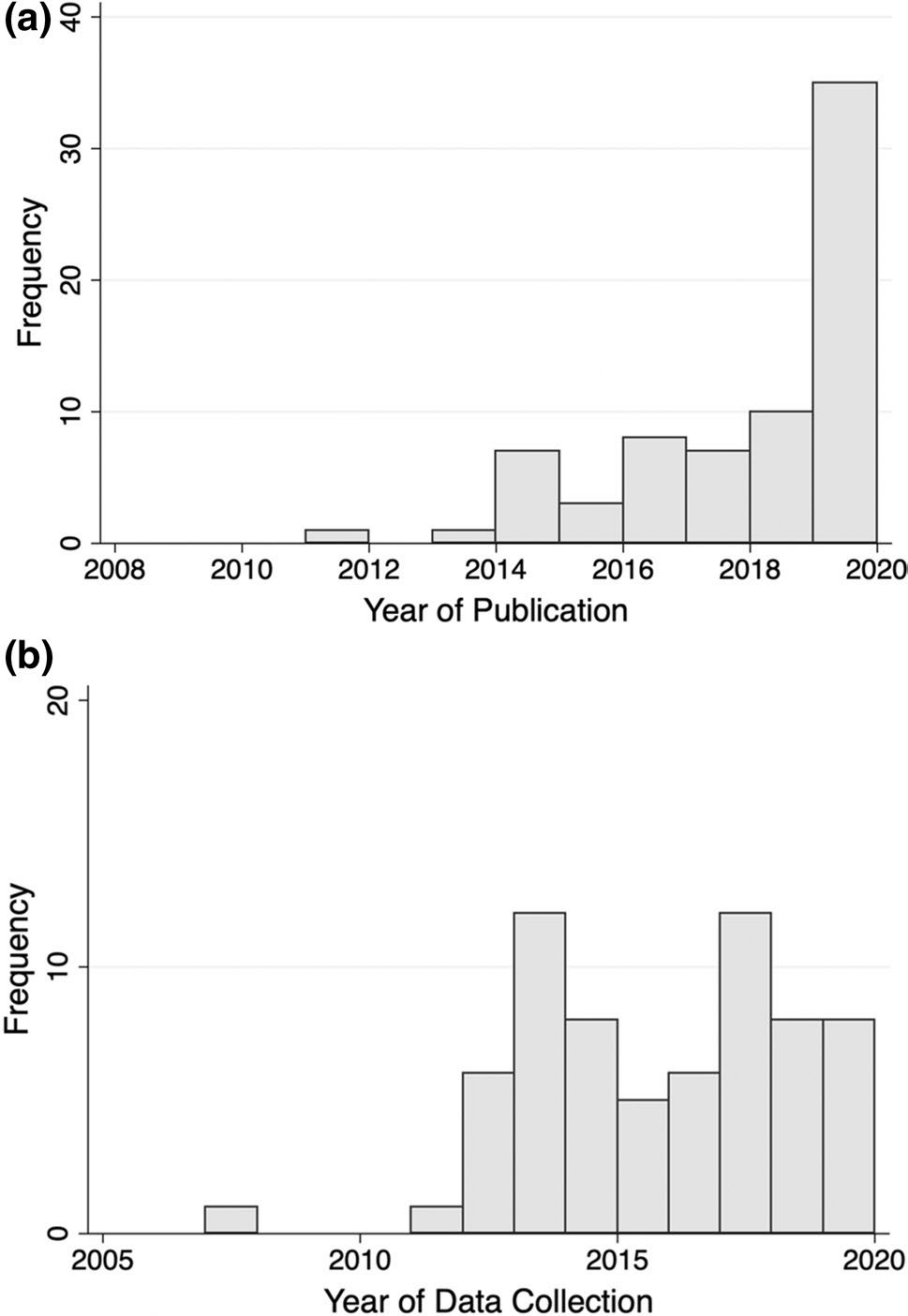
Years of publication and data collection of included studies. *Note* Year of publication is the year when the article was published or abstract presented. Year of data collection is the mid-point of the reported data collection period

A complete listing of included studies is available as *Table S1*. The number of published studies on TasP/U = U beliefs has increased in recent years (Fig. 2). The studies in our review presented information on TasP/U = U awareness (n = 39), knowledge (n = 31), attitudes (n = 44), acceptability (n = 34), and the impact of TasP/U = U information (n = 4) (Table 1). A majority of studies (n = 46, 64%) presented data from North America, Europe, or Australia; nearly all of these studies focused on men who have sex with men and other sexual minority populations. The review identified 17 studies from Africa, focusing primarily on heterosexual populations. Globally, 19% of studies included providers, 33% of studies included people receiving HIV care, and 68% of studies collected data from community-based samples. Of the community samples, 4% included people with HIV only, 14% included people without HIV only, 35% included people with and without HIV, and 45% did not collect data on HIV status, did not report HIV status, or collected data from participants with unknown HIV status. The literature reviewed included 40% qualitative studies (n = 29) and 61% quantitative studies (n = 44), with one study using both qualitative and quantitative methods. Most studies used respondent-driven, voluntary, or purposive samples, with only (n = 9, 13%) using statistically representative sampling strategies (e.g. random sampling) from an underlying population.

**Table 1:**
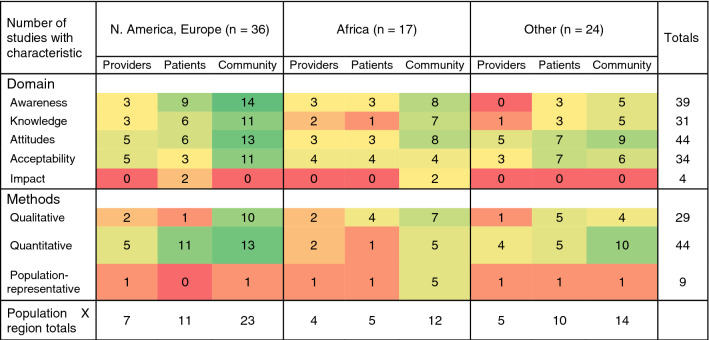
Characteristics of included studies by region and study population, Jan 2008–Oct 2020, (n = 72)

Color scheme illustrates cells where there are more studies (green) vs. fewer (red). Domains, methods, and population classifications are defined in the methods section. Studies may appear in multiple domains, populations, and regions, and therefore the row and column totals do not reflect the sum of the cells

### Awareness and Knowledge of TasP/U = U Among HIV Patients and Community Members

#### Increasing awareness among MSM in Europe, Asia, Oceania, and the Americas

Outside of sub-Saharan Africa, studies of MSM have reported high levels of awareness but also significant knowledge gaps. In an online survey conducted in 2016–2017 in New York City, 94% of 732 MSM were aware of TasP, yet just 39% thought ARVs offered “a lot” or “complete” protection against transmission [25]. In a 2015 online survey, just 27% of U.S. MSM perceived the risk of transmission when virally suppressed as close to zero [26]. In a 2012–2014 survey of 719 MSM in Vancouver (Canada), 69% of participants with HIV were aware of TasP, but just 14% provided "complete" definitions, linking TasP to ART use, viral suppression, and prevention of transmission [27]. In a 2012–2014 study in Australia, 85% of people with HIV and not on ART were aware of TasP [28]. In a 2018 survey of over 10,000 MSM with HIV in Latin America, 74% were both aware of and knowledgeable about U = U [29].

There were few studies of non-MSM populations in high-income countries, but the evidence suggests lower awareness than among MSM. In a survey of women living with or at risk for HIV in the U.S. Women's Interagency HIV Study in 2014–2015, fewer than 2% of respondents mentioned TasP as an effective HIV prevention strategy [30]. Among 520 PLHIV in Paris (France) in 2014, 94% of MSM were aware of the impact on ART on HIV transmission, compared to 83% of heterosexual men [31]. An online survey of people without HIV in Italy found that 33% of behaviorally at-risk heterosexual respondents were aware of TasP, compared to 42% of MSM [32]. Just 57% of people with HIV at a hospital detox unit in New York City believed a TasP message was accurate [33].

The literature suggests increasing awareness and knowledge over time. Between 2013 to 2015, the percentage of Australian MSM living with HIV who reported that treatment prevents transmission increased from 10 to 46% [34]. In a 2017–2018 online survey of over 100,000 sexual minority men in the U.S., the share that perceived TasP to be effective increased by 1–2% per month during the study period [35]. In a 2019 survey of 2389 people on HIV treatment in 25 predominantly high-income countries, 88% had discussed U = U with a provider or were aware that treatment reduces transmission [36].

#### Low awareness in Sub-Saharan Africa

Fewer studies have assessed knowledge and awareness of TasP/U = U in Africa. However, the available literature suggests limited diffusion of information in HIV-endemic regions. Focus groups with community members in Zambia and South Africa in 2012–2013 revealed that most participants were unaware of TasP and thought separately about treatment and prevention [37, 38]. Interviews in 2015 with South African men with and without HIV revealed that none of the participants was aware of the prevention benefits of ART [39]. In focus groups and interviews with female sex workers in South Africa, few understood the rationale behind TasP [40].

Quantitative surveys in African settings have found similar results. In a community RCT in rural Malawi in 2013, 65% of survey participants in control-group communities perceived that ART had no impact on transmission risk [41]. In a 2017 survey of young adults in rural South Africa, participants perceived a 75% annual risk of HIV transmission in a mixed status couple where the PLHIV was on ART and virally-suppressed. (The true risk is zero.) [42]. Similar results were found among university students in urban South Africa [43]. Consistent with low knowledge of TasP, a survey in rural Uganda found that men perceived that it was very unlikely a couple could have different HIV statuses, even if the HIV-positive partner was on ART [44].

Some recent studies indicate higher knowledge. In a 2020 study of men presenting for HIV testing in a peri-urban community in South Africa, 78% reported that people with undetectable VL could not transmit HIV [45]. Higher levels of awareness have also been documented among mixed-status couples accessing HIV care—a group that has been prioritized for TasP/U = U messaging. In interviews conducted with mixed-status couples in Kenya and Uganda from the Partners Demonstration Project (PDP) in 2017, participants reported that ART lowers transmission risk but lacked a full understanding of viral suppression. [46].

#### Differential Knowledge and Awareness by HIV Status

Information on TasP has diffused more rapidly to PLHIV, compared to people without HIV. Among MSM in Vancouver, 2012–2016, about two-thirds of PLHIV were aware of TasP, compared to just one third among people without HIV or of unknown status [27, 47]. A 2014 online survey (n = 3596) in Italy found that 61% of HIV-positive participants and 42% of HIV-negative MSM participants were aware of TasP [32]. A 2012 online survey in Australia reported that 52% of MSM with HIV and 15% of MSM without HIV believed “transmission is unlikely when an HIV-positive man was taking ART” [48]. In a 2016 study in Australia, 20% of MSM with negative or unknown HIV status agreed that “a person with an undetectable viral load cannot transmit HIV” [49]. In a 2019 online survey in Brazil, 79% of PLHIV rated U = U as completely accurate, but just 44% of HIV-negative sexual minority men and 17% of general population participants rated U = U as completely accurate [50]. In a 2017 survey in the U.S., MSM without HIV or with unknown status were less likely than HIV-positive MSM to report understanding the concept of undetectable viral load [51]. In a 2017–2018 survey of over 100,000 U.S. sexual minority men, 51% of men with HIV, 19% of men without HIV, and 11% of status-unknown respondents rated U = U as “completely accurate” [35]. In a 2018–2019 survey of HIV-negative MSM in the U.S. mid-Atlantic region, just 38% viewed the message as completely accurate [52]. In interviews conducted during 2018–2019 with HIV-negative U.S. MSM engaged in exchange sex, 72% perceived TasP to be effective [53].

#### Summary

A complete summary of the evidence on awareness and knowledge is available as **Table S2**. Awareness of TasP/U = U is widespread among MSM living with HIV in high-income countries. However, knowledge gaps remain regarding the perception that U = U is “completely accurate”. Awareness and knowledge are considerably lower among MSM without HIV or with unknown status. The extant literature suggests lower knowledge and awareness of TasP among populations residing in HIV-endemic regions in sub-Saharan Africa and among non-MSM populations outside of sub-Saharan Africa. The absence of widespread, shared understanding of TasP by people with and without HIV may limit uptake of TasP as a prevention strategy [54].

### Attitudes and Acceptability of TasP Among HIV Patients and Community Members

#### Skepticism of the Science Linked to Low Acceptability

A key measure of acceptability is the extent to which people are willing to rely on TasP as a substitute for other prevention methods. Among HIV-negative gay men in Australia, interviewed in 2012, just 10% reported they would rely on TasP alone to prevent HIV [55]. In a later analysis of the same sample, 92% worried that ART does not completely eliminate transmission risk, and 90% preferred to use condoms if their HIV-positive partner was on ART [48]. In other Australian studies, 83% of participants were uncertain or critical of TasP, citing skepticism of TasP science and potential behavioral risks [28]; and just 48% were confident in using U = U [56].

In Uganda and Kenya in 2017, people in mixed-status couples reported doubts about the effectiveness of TasP or their partners adherence to ART, and in turn preferred to use condoms or PrEP [46]. In Kenya, 2017–2019, HIV-negative partners of PLHIV expressed similar skepticism and reported they would still use PrEP and condoms even if their partner was virally suppressed [57]. Among mixed-status couples in Canada surveyed in 2017, 47% of participants agreed that "when a person's VL is undetectable, they can safely have intercourse without a condom." [58].

#### Acceptability Increased with Knowledge and Experience

**Fig. 3:**
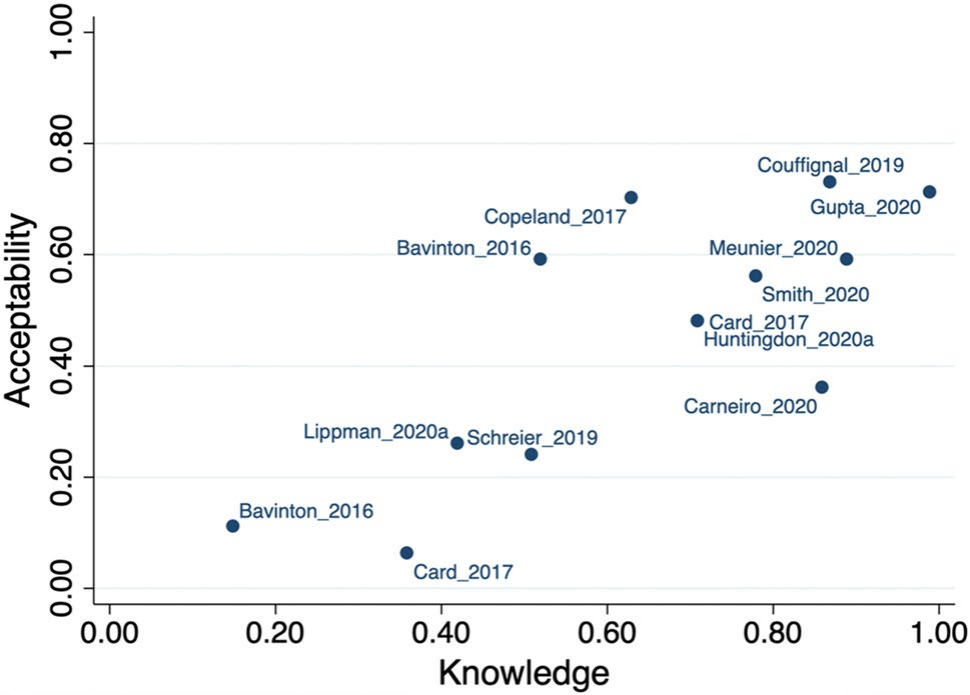
Association between knowledge and acceptability of TasP in studies that assessed both

Several studies reported measures of both knowledge and acceptability for the same sample. As illustrated in Fig. 3, higher knowledge was generally associated with higher acceptability of TasP.

In Zambia and South Africa, after researchers explained TasP, respondents were receptive to it but still had concerns about sexual risk behavior and ART uptake [37]. Men at high risk for HIV infection in Vancouver also reported high receptiveness to TasP, despite low initial awareness [59]. Once informed, people had positive attitudes towards TasP because they perceived benefits. Among mixed status couples interviewed in 2013 in Kenya, participants preferred TasP over PrEP, citing the direct health benefits of TasP for PLHIV, a belief that medication should be given first to PLHIV, and perceived limited motivation of HIV-negative people to take ARVs. Participants cited the ability to have condomless sex and to conceive naturally as benefits of TasP [60].

People also gained confidence in TasP/U = U from personal experience. In Kenya, mixed status couples who had condomless sex and did not acquire HIV expressed greater confidence in U = U [57]. Similarly, in Australia, participants were initially apprehensive about relying on TasP, but concerns faded over time as they had repeated condomless sex without transmission [61].

More recent data indicate growing acceptability of TasP. In interviews conducted during 2018–2020 with HIV-negative MSM in the U.S., 59% of participants were either willing to rely on or had relied on TasP for prevention [53]. TasP was the most common prevention strategy used by mixed-status MSM couples in a recent qualitative study in the U.S. [62].

#### TasP Could Motivate ART Uptake and Adherence

Among HIV-positive women in the U.S., 87% wanted to learn more about TasP and 68% not already taking ART were likely to consider ART to prevent transmission to partners [30]. In South Africa, participants supported dissemination of TasP information and expressed that TasP knowledge would incentivize testing, treatment, and adherence [39]. Among HIV-positive study participants in Paris, 73% reported that TasP alleviated fears of transmission and 45% reported improved ART adherence with TasP [31]. In a survey of people with HIV in 25 countries, participants who had discussed U = U with their provider had lower odds of suboptimal adherence to ART [36]. We note the challenges in disentangling cause and effect, as providers may be more likely to recommend TasP/U = U to patients who are more adherent.

#### An Affirmative Way for PLHIV to Understand Their Status

Among MSM in Singapore, participants described becoming undetectable as a turning point or achievement, and reported that U = U led to improved self-image and greater ease in coming to terms with their HIV positive diagnosis. Respondents also reported a sense of liberation from fear and self-stigma and sense of equal value in serodifferent relationships [63]. Similarly, among sexually-active gay and bisexual men in Canada, many participants felt that being undetectable was more a part of their identity than being HIV-positive [64]. In a survey of MSM living in Latin America, U = U knowledge was associated with lower rates of anxiety and depression symptoms and a lower internalized homonegativity score [29]. TasP/U = U was also perceived to have potential to improve quality of life. PLHIV in Australia [65] and Latin America [66] were more likely to sexually adjust to their HIV diagnosis if they knew about U = U.

#### Barriers to Acceptability

Barriers to acceptability remain even among people knowledgeable about TasP. Traditional barriers to HIV treatment uptake and adherence [67] limit uptake. In Kenya, barriers to use of TasP included perceived side effects of ART, adherence challenges, and status acceptance [60]. In Vancouver, stigma was seen as a barrier to use of TasP, while access to accurate scientific information was a facilitator [68]. In Scotland, study participants described barriers including criminalization of transmission, increased burden of treatment, and perceptions of risk [69].

At the same time, TasP itself is not universally accepted. In Malawi, women living with HIV did not accept the prevention aspect of treatment, as it did not align with their beliefs of health and illness [70]. In South Africa and Zambia, participants viewed treatment and prevention separately, with prevention not coming to mind when discussing ART [37]. Existing mental models of HIV and ART that are not rooted in concepts of viral suppression may limit acceptance of TasP/U = U.

Finally, acceptability of TasP differs with HIV status. In-depth interviews with sexual minority men in Vancouver revealed that participants without HIV were reluctant to incorporate a partner’s undetectable HIV status into their sexual decision-making, and the authors identified enduring “sexual stigma attached to HIV” even in the context of U = U [71]. In a 2017–2018 U.S. study of HIV-negative men and trans people who have sex with men, trust in U = U was associated with greater willingness to have condomless anal sex; yet just 42% of respondents who were aware of U = U indicated they trusted it [72].

#### Summary

A complete summary of the evidence on attitudes and acceptability is available as Table S3. Acceptability of TasP has increased over time, as the science on U = U has disseminated. Higher knowledge was associated with greater acceptability of TasP across studies. Perhaps the greatest barrier to acceptability is lack of knowledge in many populations globally with high HIV prevalence. In some settings, disseminating information alone may be insufficient to change norms, as TasP may challenge fundamental beliefs about HIV and ART. Further, people without HIV expressed concerns about relying on the adherence behavior of HIV-positive partners for protection, suggesting an important limitation of TasP.

### Knowledge and Attitudes Among Health Providers

#### Knowledge Gaps Among Providers

Gaps in knowledge persist among HIV service providers. In a 2017 survey of participants in continuing medical education with the International Antiviral Society-USA (IAS-USA), just 51% were aware that condomless sex does not lead to HIV transmission in the setting of viral suppression [73]. In a 2012–2014 survey of non-medical HIV service providers, just 63% “strongly agreed” that “suppressing HIV viral load with ART reduces risk of transmitting HIV” [74]. In a 2017–2018 survey of stakeholders in HIV services, including providers, advocates, and patients in New York State, 84% of participants were aware of U = U but only 58% were confident in the concept [75].

Provider knowledge gaps may lead to ineffective communication on TasP. For example, a study in Kenya interviewed both health providers and HIV-negative members of mixed status couples using PrEP in 2017–2018. Heath providers were aware of TasP/U = U but reported incomplete knowledge and inconsistent beliefs: for example, some believed—inaccurately—that U = U only worked with consistent condom use. In turn, members of mixed HIV status couples reported they were informed about U = U by the providers, but that they did not believe the message [76].

#### Provider Attitudes and Acceptability

Providers reported mixed attitudes about TasP, although most were supportive. In a 2014 international survey of health providers, 17% perceived that other providers were opposed to TasP and 37% were unsure [77]. Some providers expressed hesitance to share information about TasP for fear that it could lead to STIs or undesired pregnancy, or HIV transmission if people rely on TasP without viral suppression. Others worried that TasP would lead to more condomless sex among PLHIV—often referred to as “sexual disinhibition” in this literature—which they perceived as a negative outcome despite the absence of transmission risk during condomless sex while virally suppressed [77, 78].

In a 2017 U.S. survey, 76% of providers commonly or always recommended condoms to patients with viral suppression, and just 3% of medical providers agreed that condomless sex in the setting of full viral suppression with good adherence could be recommended as “settled science” [73]. In Malawi, HIV care providers and program stakeholders expressed concerns related to: equating ‘undetectable’ with ‘healed’, which may impact adherence negatively, and to a potential increase in promiscuity and HIV re-infection [79]. Kenyan health providers reported fears that telling patients about U = U would lead to other risk behaviors, or that consequent HIV transmission would be blamed on them. Observations of fluctuating viral loads among their patients reduced their acceptance of U = U [57]. IN 2013, HIV nurses in the U.K. perceived that TasP offered benefits including reassurance for patients that loved ones are protected; however, they expressed concerns about “sexual disinhibition” [80]. Stigma around HIV, including associations with promiscuity and deviance, associations with homosexuality, and racialized stigma may also limit provider acceptance and communication on TasP/U = U [18, 81].

Acceptability of TasP among providers has increased over time as evidence of clinical benefits of early ART and the science of U = U have become clear. In 2012, prior to the START [8] and TEMPRANO [9] trials showing clinical benefit of early ART, some providers hesitated to endorse TasP out of fear that public health concerns might be put ahead of the benefits to individual patients [82]. In a 2018 survey of members of the British Medical Association, 71% of providers reported that they raise the subject of U = U routinely with HIV patients. However, most providers did not accurately convey that the risk of transmission was “zero”, instead using more ambiguous language such as “negligible” or “extremely low” [83]. A study of reproductive health providers in Brazil found that 96% “strongly agreed” or “agreed” that they would encourage a mixed status couple with undetectable HIV to attempt natural conception [84].

### Impact of Disseminating Information on TasP/U = U

While a large number of studies have documented rising knowledge and acceptability of TasP/U = U, very few studies have rigorously evaluated the impact of disseminating TasP information on behaviors and clinical outcomes. Table 2 describes the characteristics of the four studies that met our inclusion criteria for impact evaluations of interventions disseminating information on TasP/U = U. Two of the studies were intensive behavioral interventions for PLHIV tested in the U.S. Another study tested U = U messaging to recruit men into HIV testing in South Africa. The fourth was a large community-randomized trial of a community-education intervention in Malawi.

**Table 2:** Impact evaluations of interventions disseminating information on TasP/U = U

Study	Intervention description	Comparison	Study design	Outcome measure	Result description
Kalichman et al. (2011)	Intensive behavioral intervention for PLHIV: Integrated risk reduction and adherence intervention, with information provided on HIV transmission and viral load. Intervention informed by conflict theory of decision making	Attention-placebo control: support group for individuals living with HIV/AIDS	436 participants randomized to treatment or control; followed up via monthly telephone-based pill counts and surveys at 3, 6, and 9 months	Medication adherence; condomless sex with HIV-negative partners; and self-reported STIs; beliefs about TasP	The intervention led to increased ART adherence, less condomless sex with HIV-negative partners, and fewer new reported bacterial STIs. The proportion of ART taken was approximately 10 percentage points higher in the intervention group relative to the control group over the follow-up period (p < 0.05)
Kalichman et al. (2018)	Mobile health behavioral intervention for PLHIV: One in person workshop and four phone sessions addressing TasP, ART adherence, access to care, sexual decisions, and other health topics. Informed by Social Cognitive Theory	Attention-placebo control: sessions focused on HIV-unrelated health goals and behaviors	500 participants randomized to treatment or control; biomarkers collected at 12 months; monthly pill counts and 3-monthly surveys	HIV viral load; genital tract inflamation; medication adherence; condomless sex with HIV-negative partners; self-reported STIs; TasP beliefs	Intervention group had lower viral load and greater ART adherence, with no increase in reported STIs or genital tract inflamation. Relative to controls, the intervention group had significantly lower 12-month viral load (5326 vs. 11,914 copies/μL, OR 0.56, p < 0.01)
Smith, et al. (2020)	Recruitment materials for HIV testing: Flyer and script recruiting men to HIV testing at local testing centers using language about U = U/TasP. Informed by behavioral economics theory on framing	Attention placebo control: flyer and script recruiting men to HIV testing at testing center, no U = U message	1048 men were invited for HIV testing over 12 days each at 5 mobile testing sites; 180 men tested and participated in a survey	Participation in same-day HIV testing	In the SOC group, 13% tested for HIV. In the U = U group, 22% tested for HIV. Men in the U = U group had greater odds of getting tested for HIV (aOR 1.58, 95%CI 0.98, 2.57), (p < 0.1)
Derksen et al. (2020)	Community health education meeting: provided information on both health and prevention benefits of ART and about transmission risks with and without ART. Informed by microeconomic model in which community knowledge of TasP reduces HIV stigma	Attention-placebo control: health education meeting giving information just on the health benefits of ART	122 villages stratified and randomly assigned to intervention vs. control	Uptake of HIV testing at local clinics; survey at 4-months after baseline (n = 1358)	In post-intervention survey, 19% of controls and 80% of intervention group chose ART as an HIV prevention method. The intervention reduced discriminatory beliefs and increased uptake of HIV testing by over one-third, from 0.56 to 0.76% per month at community level (p < 0.05)

Kalichman et al. [85] tested an intensive behavioral intervention for PLHIV designed to integrate information on TasP with adherence support and counseling on sexual risk reduction, in order to reduce onward transmission of HIV. The study randomized 436 PLHIV in Atlanta, U.S., to the intensive behavioral intervention or to an attention placebo control condition. The intervention consisted of two one-on-one counseling sessions and five two-hour group sessions on topics including HIV transmission, sexual decision-making in the context of detectable and undetectable viral load, sexual decision-making and substance use, and ART adherence to improve health and reduce infectiousness. The control condition was an “attention placebo” with a similar number of sessions on health topics unrelated to HIV. In unannounced pill counts over the subsequent year, the intervention group had significantly higher adherence than the control group (p < 0.05). The intervention group reported no more condomless sex with HIV-negative partners than controls and actually reported fewer new bacterial STIs (3.5% vs. 8.6%, p < 0.05).

Building on the 2011 study, Kalichman et al. [86] evaluated a mobile health behavioral intervention, which adapted the prior facility-based intervention to be less intensive, more accessible, less costly, and easier to scale. The study randomized 500 PLHIV in Atlanta to the intervention or attention placebo control. People in the intervention arm participated in one in-person group workshop and in four phone-based sessions addressing TasP, ART adherence, access to care, sexual decisions, and other health topics. HIV viral loads and biomarkers for genital tract inflammation (GTI) were collected at 12 months. Monthly unannounced pill counts and 3-monthly surveys of risk and adherence behaviors were also conducted. Relative to controls, the intervention group had significantly higher adherence (p = 0.04), significantly lower 12-month viral load (5,326 vs. 11,914 copies/μL, OR 0.56, p < 0.01), and similar rates of GTI symptoms, STI diagnoses, and GTI biomarkers. Together, the two studies by Kalichman et al. illustrate that TasP information can be leveraged to improve ART adherence and reduce viral load, without adverse consequences for STI incidence.

Two studies conducted in sub-Saharan Africa suggest that “light touch” messaging on U = U can also motivate care-seeking behaviors, highlighting the importance of information itself. Smith et al. [45] conducted a pilot trial of recruitment materials for HIV testing that emphasized U = U. The intervention, developed through a community-participatory process, sought to increase HIV testing among men living in Mitchells Plain, South Africa. Health workers at mobile testing sites went into the community to recruit men to test for HIV. On clinic days randomized to the intervention, health workers used a flyer and script that emphasized TasP/U = U. On clinic days randomized to control, the health workers used a standard of care script. The pilot, conducted in March 2020, was stopped early due to COVID-19. During 12 days of recruitment, 1048 men received invitations for HIV testing at 5 mobile testing sites. Of these, 180 men tested and participated in a survey. In the U = U group, 22% of men invited were tested for HIV and 6% tested positive. In the control group, 13% of men invited tested for HIV and 4% tested HIV-positive. The adjusted OR for testing was 1.58 (95% CI 0.98, 2.57).

Whereas the above studies provided information on TasP at the individual-level, the full benefits of TasP may only be realized once other community members (including prospective sex partners) are aware that ART leading to viral suppression eliminates transmission risk. Comfort disclosing one’s status and discussing TasP may depend on potential partners’ knowledge of TasP. Further, community beliefs may shape care-seeking behaviors among those who think that they might have HIV but fear discrimination from potential sex partners. Community-level awareness of TasP or U = U could shift perceptions of someone using ART from being a high-risk partner to a low- or no-risk partner, leading to greater HIV testing and ART uptake.

To assess this pathway, Derksen et al. [87] conducted a large cluster-randomized trial of a community-level TasP education intervention in rural Malawi. A single community meeting was conducted in 122 villages. In treatment villages, educators provided information on both the health and prevention benefits of ART, using interactive techniques to teach about transmission risks with and without ART. In control villages, educators provided information on only the health benefits of ART. The intervention led to sharp changes in beliefs about TasP. In a household survey (n = 1358) across study villages, 80% of people living in treatment villages vs. 19% of people in control villages mentioned ART as a prevention strategy. Although uptake of HIV testing was low in both groups (an annual rate of 7%), the authors found a 36% higher rate of testing among people living in intervention communities. Further, HIV testing depended primarily on people’s perceptions of TasP beliefs in the community, not on their own beliefs or their spouse’s beliefs. Discriminatory beliefs towards people with HIV also fell in treatment communities. The percent preferring “untested” partners to “HIV positive partners on ART” was 14 percentage points lower in treatment relative to control villages, for example, and the percent believing that a person using ART would not find a new partner was 11 percentage points lower in intervention villages. These findings illustrate that stigma based on the fear of HIV acquisition continued to be prevalent in rural Malawi, that this stigma discouraged HIV care-seeking, and that TasP information helped to alleviate this stigma and increase care-seeking.

## Discussion

More than 20 years after the risk of HIV transmission was directly correlated with plasma viremia [1], a decade after the HPTN-052 trial showed that HIV treatment is among the most effective HIV prevention strategies [3], and years after large cohorts established sexual HIV transmission risk to be zero in the context of sustained viral suppression [4–6], information on TasP and U = U has yet to reach many HIV-endemic populations. We conducted a systematic review to assess levels of awareness, knowledge, attitudes, and acceptability of TasP/U = U, and impacts of interventions sharing this information.

### Our Review had Four Key Findings

First, awareness regarding TasP/U = U has increased over time, but in-depth knowledge and belief in the scientific evidence remains uneven. Information on TasP/U = U has diffused fastest among MSM populations in Europe, Asia, Oceania, and the Americas, but disbelief is still widespread in these communities, particularly among people who are HIV-negative. Large gaps in knowledge persist in Africa where 2 out of every 3 PLHIV reside. Second, once people believed the science on TasP/U = U, acceptability of TasP was generally high. Among PLHIV, U = U was viewed as enabling a positive self-image and reduced internalized stigma. Among people without HIV, not trusting partner adherence was a barrier to acceptability of TasP. Third, interventions disseminating TasP/U = U information have had beneficial impacts on HIV testing, adherence, viral suppression, and stigma reduction, without leading to increased STI incidence. Fourth, not all health providers are well-informed about TasP/U = U and some have judgmental views which make them unwilling to share information about TasP with their patients.

### Our Review Identified Gaps in the Literature

Out of the 72 studies identified in our review, only 17 studies were conducted in Africa and just 5 of these were quantitative studies with population-representative samples. Further research on population knowledge and attitudes related to TasP will be important to guide the implementation of U = U interventions in sub-Saharan Africa. We also found only 4 rigorous studies on the effect of TasP/U = U messaging globally. More research is needed to assess the impact of different types of TasP/U = U interventions (intensive vs. light-touch), implemented in different contexts (from the counselling room to the street-corner), delivered by different people (nurses vs. pastors vs. peer educators), in populations with different sexual behaviors, in different health-systems contexts, in cultures with different mental models of disease transmission, implemented at different levels (e.g. individual vs. couple vs. community), targeting different theoretical pathways to impact (e.g. self-image [63] vs. HIV prevention altruism [88] vs. community stigma [41]), as well as long-term impacts on viral suppression, mental health, and STI incidence.

We identified protocols in PubMed for two ongoing intervention trials of TasP/U = U interventions among Black and Latino MSM in the U.S. [89, 90], as well as one cluster-randomized trial in South Africa that attempted to shift knowledge, norms, and behaviors related to TasP through a 3-year community-mobilization intervention [91]. A search of active trials on clinicaltrials.gov (HIV AND (TasP OR U = U)) revealed one additional study of an intervention to integrate U = U messaging into HIV counseling in South Africa (NCT04504357). These four studies alone have the potential to double the available information on TasP/U = U interventions, highlighting the thinness of the current evidence base.

### Our Review had Several Limitations

First, we may have inadvertently excluded relevant studies that did not include search terms related to TasP or U = U in the title or abstract. We also may have missed studies that were not published in a journal indexed in PubMed, presented at one of the three included conferences, or referred to us via personal communication. We chose our search terms because they are the most widely used descriptors of the scientific evidence linking HIV treatment to lower transmission risk. We believe it is unlikely that many recent studies would have addressed these topics without mentioning these concepts.

Second, per our inclusion criteria, we excluded studies that did not explicitly assess TasP/U = U awareness, knowledge, attitudes, acceptability, or the impact of TasP/U = U information. Among excluded studies were several high-profile community-based randomized trials of test-and-treat strategies, including PopART, SEARCH, ANRS-TasP, Ya Tsie, and MaxART, in which messaging on TasP was not the primary focus. For example, even after the ANRS-TasP trial in South Africa, community members were unaware of the prevention benefits of ART [54]. We also did not include studies on factors related to the feasibility of U = U, such as persistent viremia, prevalence of STIs [16], and people’s beliefs about their own viral suppression [17].

Third, given the diversity of study methods we were not able to rate the quality of studies beyond a few crude measures, e.g. sample size, whether the sample was representative, and whether causal inferences could be made. Fourth, the included studies used a wide range of scales and elicitation methods, preventing simple summaries of the data. To enable comparisons across settings, future studies should collect quantitative assessments of perceived transmission risk (with and without TasP) in addition to qualitative endorsements of statements about TasP/U = U [35, 41, 42].

Fifth, most existing studies of impact delivered TasP/U = U messaging alongside other interventions such as enhanced adherence counseling [85, 86] or enhanced messaging around other benefits of modern single-pill, low-toxicity ARVs [45]. Just one study enabled inferences on the impact of TasP/U = U messaging alone [87]. These multimodal interventions correctly diagnose that knowledge and attitudes regarding TasP are precursors and foundations of behavioral intent, but are not sufficient to cause behavior change. Understanding what complementary factors facilitate TasP uptake in the context of rising knowledge is an important avenue for future research.

### Conclusion

The science of TasP has transformed global HIV policy, with countries worldwide embracing test-and-treat and many now engaging in public information campaigns about TasP/U = U. Our review finds that there remain large gaps in lay knowledge about TasP, particularly in Africa and particularly among people without HIV. Our findings also indicate that disseminating information on TasP/U = U could increase HIV testing, reduce stigma, and improve HIV treatment outcomes, leading to better health for PLHIV and lower risk of transmission to others.

## Supplementary Information

Below is the link to the electronic supplementary material.

Supplementary file1 (XLSX 528 KB)

## Data Availability

All manuscripts reviewed are in the public domain. Extracted data are available as supplementary tables.
